# Water intoxication presenting as maternal and neonatal seizures: a case report

**DOI:** 10.1186/1752-1947-2-366

**Published:** 2008-12-04

**Authors:** Timothy H Chapman, Mark Hamilton

**Affiliations:** 1 Department of Intensive Care, St Georges Hospital, London, SW17 0QT, UK

## Abstract

**Introduction:**

We present an unusual case of fitting in the mother and newborn child, and the challenges faced in the management of their hyponatraemia due to water intoxication.

**Case presentation:**

A previously well 37-year-old, primigravid Caucasian woman presented with features mimicking eclampsia during labour. These included confusion, reduced consciousness and seizures but without a significant history of hypertension, proteinuria or other features of pre-eclampsia. Her serum sodium was noted to be low at 111 mmol/litre as was that of her newborn baby. She needed anti-convulsants with subsequent intubation to stop the fitting and was commenced on a hypertonic saline infusion with frequent monitoring of serum sodium. There is a risk of long-term neurological damage from central pontine myelinolysis if the hyponatraemia is corrected too rapidly. Mother and baby went on to make a full recovery without any long-term neurological complications.

**Conclusion:**

There is little consensus on the treatment of life-threatening hyponatraemia. Previous articles have outlined several possible management strategies as well as their risks. After literature review, an increase in serum sodium concentration of no more than 8–10 mmol/litre in 24 hours is felt to be safe but can be exceeded with extreme caution if life-threatening symptoms do not resolve. Formulae exist to calculate the amount of sodium needed and how much hypertonic intravenous fluid will be required to allow safer correction. We hypothesise the possible causes of hyponatraemia in this patient and underline its similarity in symptom presentation to eclampsia.

## Introduction

Water intoxication before labour is an unusual but documented cause of fitting in the immediate postpartum period. It tends to be associated with iatrogenic fluid overload, prolonged administration of oxytocin or psychiatric disorders [1]. The risk of water intoxication may be due to a culmination of increased body water in pregnant women, the birth-related activation of hormonal systems along with the mother drinking too much water during or in the run up to labour. In infants, it tends to be associated with bottle-feeding with diluted formula or water. Awareness of the diagnosis is important because it mimics pre-eclampsia or dehydration [2].

## Case presentation

This is the case of a 37-year-old, primigravid woman who underwent spontaneous vaginal delivery. She was deemed to be well before labour with an uncomplicated pregnancy having walked to the delivery unit that morning. She had a normal blood pressure throughout pregnancy as well as in the postpartum period.

During labour, it was noticed that the patient was becoming increasingly confused but hours later she underwent normal vaginal delivery. She was however noted to be still confused by her husband. Twenty-five minutes after delivery, her first generalised tonic-clonic seizure occurred, lasting about 5 minutes before spontaneously resolving. Despite being given magnesium sulphate for possible eclampsia at that time, she remained confused and had a further seizure within 3 hours. A further infusion of magnesium was administered unsuccessfully, thus the patient was given intravenous anti-convulsants causing a decrease in consciousness requiring subsequent intubation for airway protection. She had a one-off elevated blood pressure of 160/102 during fitting, and at all other times she was normotensive. Her urinalysis showed a trace of protein after delivery. Eclampsia was subsequently thought to be unlikely. At the time of the mother's second fit, the newborn baby also had a seizure. Further information from the partner suggested that the couple normally drank a lot of water between them, with the mother drinking up to 4 litres of water a day. The mother had recently been drinking more than this because of the recent hot weather. She also continued to drink increasing amounts of water in the run-up to labour due to a feeling of thirst, after being taught to avoid dehydration in antenatal classes. Liberal fluid intake is encouraged to counter the fluid losses and energy expenditure during childbirth [2]. There was no other medical or drug use history. The patient had an otherwise normal healthy diet.

Her immediate blood tests showed a metabolic acidosis, likely to have been caused by the two fits, as well as low serum sodium of 111 mmol/litre, low urea of 0.8 mmol/litre along with low chloride and potassium levels. Urinalysis revealed a urine osmolality of 67 mosmol/kg and urinary sodium of 10 mmol/litre. A paired serum osmolality of 228 mosmol/kg was consistent with a dilute serum and urine, suggesting water overload. Neurological examination before intubation showed normal fundi and no focal neurological abnormality other than the marked confusion. The newborn baby also had low serum sodium of 108 mmol/litre, urine osmolality of 46 mosmol/kg, urinary sodium <10 mmol/litre and serum osmolality of 225 mosmol/kg. The mother was subsequently managed on the intensive care unit and the baby on the special care baby unit. A maternal lumbar puncture was normal as was subsequent cranial magnetic resonance imaging (MRI) showing no venous sinus thrombosis or evidence of central pontine myelinolysis.

The maternal sodium was initially corrected at a rate of 1 to 2 mmol/l/hour with hypertonic saline. This rapid correction was done due to her ongoing seizure risk and stopped when the serum sodium reached 125 to 130 mmol/litre (Table 1) or the patient deemed to be no longer at risk of life-threatening manifestations of severe hyponatraemia with cessation of seizure activity. Too rapid a correction of serum sodium can trigger demyelination of pontine and extrapontine neurons to occur after one or up to several days after the correction. This causes neurological dysfunction, including quadriplegia, pseudobulbar palsy, seizures and death. Most reported cases of osmotic demyelination have occurred after rates of correction exceeding 12 mmol/litre per day [3]. A correction rate of up to 8 to 10 mmol/litre per day is recommended to reduce the risk of osmotic demyelination but can be cautiously exceeded if severe symptoms do not respond [3,4]. Due to the acute and life-threatening nature of her illness, hypertonic saline was chosen to raise the serum sodium rather than fluid restriction which may be more appropriate in more chronic conditions. The amount of hypertonic saline needed is estimated by calculating the sodium deficit using the following equation:

**Table 1:** Serum and urine electrolyte/osmolality results during treatment

	Day 1	Day 2	Day 3	Day 4	Day 7	Day 14
Sodium (135–145 mmol/litre)	111	123	128	131	137	138
Potassium (3.5–4.7 mmol/litre)	3.3	4.3	4.4	4.0	3.7	4.6
Chloride (98–109 mmol/litre)	86	97	103	104	104	104
Urea (2.5–8.0 mmol/litre)	0.8	1.1	1.9	2.4	2.1	1.6
Serum osmolality (280–300 mosmol/kg)	228	248	267	271		287
Urine osmolality (100–1400 mosmol/kg)	67	80	141			
Urine sodium (mmol/litre)	10

Sodium deficit = total body water × (desired Na^+ ^- actual Na^+^)

Total body water is estimated as lean body weight times 0.5 for women or 0.6 for men.

This gave us an estimate in mmol/litre of sodium required, thus allowing a suitable volume of hypertonic saline to be infused to achieve the above rate of correction [3]. Another equation can also be used to see the effect of 1 litre of any intravenous solution on serum sodium.

Change in sodium = (infusate Na^+ ^- actual serum Na^+^) ÷ (total body water + 1)

Frequent sodium measurements are still required to assess the efficacy of treatment. Blood tests in both mother and baby subsequently slowly normalised. This improvement allowed the mother to be extubated the next morning. It was noted that the mother had fractured her left neck of femur as well as having a right shoulder fracture dislocation, both of which were subsequently repaired without incident. Bone densitometry and biopsy showed osteoporosis. A short synacthen test was normal ruling out primary adrenal failure as a possible cause for the hyponatraemia and osteoporosis. Parathormone and thyroid function testing was also normal. The osteoporosis could be associated with the pregnancy, as no other cause was identified in this young primigravid woman. The mother subsequently made a full neurological recovery with no further episodes of confusion or fitting.

## Conclusion

The overriding cause of fitting in this patient is arguably from hyponatraemia due to psychogenic polydipsia. The confusion and fear of dehydration combined with a high normal fluid intake caused the patient to drink even more water than before, thus developing a vicious circle of polydipsia and worsening water intoxication. Symptoms are more severe with an acute reduction in serum sodium and occur due to cerebral oedema. The earliest findings are typically nausea and malaise, followed by headache, lethargy and confusion. Eventually seizures, coma and respiratory arrest will follow. Overly rapid correction may also be hazardous causing central pontine myelinolysis, seizures, paraesthesiae, and striatal syndrome among others [3,4]. Seizures in the newborn are recognised as the fetal plasma sodium level mimics the maternal plasma sodium level across the placenta. The fetal plasma sodium decreases slowly in response to acute reductions in maternal plasma sodium, eventually achieving equilibrium. Thus, the fetal plasma sodium level will mirror maternal hyponatraemia [5].

The natural release of oxytocin hormone during labour with its similarity to anti-diuretic hormone (ADH) may also be a factor. Excessive ADH is recognised to cause hyponatraemia, and there have been reports [6] of water intoxication due to intravenous oxytocin administration in otherwise normal pregnant women. These have all involved oxytocin administration though none have looked at intrinsic oxytocin effects on sodium levels during labour. It is of note that there was no intravenous administration of oxytocin during this labour.

This patient presented with symptoms very similar to those of eclampsia, the initial treatment of which was unsuccessful. It is therefore important to consider other causes of fitting when dealing with these cases, and be aware of the effect of maternal pathology on the newborn which may share a common aetiology.

## Consent

Written informed consent was obtained from the patient for publication of this case report and any accompanying images. A copy of the written consent is available for review by the Editor-in-Chief of this journal.

## Competing interests

The authors declare that they have no competing interests.

## Authors' contributions

TC had direct participation in management of the case, collected and analysed data, and drafted, revised and referenced the manuscript. MH had direct participation in management of the case, was involved in critical revision of the manuscript and revised the manuscript. Both authors read and approved the final manuscript.
